# Monochorionic Twins: A Delicate Balance

**DOI:** 10.3390/jcm8101711

**Published:** 2019-10-17

**Authors:** Enrico Lopriore, Liesbeth Lewi, Asma Khalil

**Affiliations:** 1 Department of Neonatology, Leiden University Medical Center, J7-48, Albinusdreef 2, 2333 ZA Leiden, The Netherlands; 2 Department of Obstetrics and Gynaecology, University Hospitals Leuven, Leuven, Belgium; liesbeth.lewi@uzleuven.be; 3 Fetal Medicine Unit, St George’s Hospital and St George’s, University of London, London, UK; akhalil@sgul.ac.uk

Monochorionic (MC) twins are identical twins who share one placenta, with vascular anastomoses connecting the circulations of both fetuses. Although the occurrence of MC twin pregnancies is rare (only 0.4% of the general population), associated complications due to a shared placental circulation are frequent and may be very severe [1]. MC twins are at increased risk of perinatal morbidity and mortality, due to the development of twin-twin transfusion syndrome (TTTS), selective fetal growth restriction (sFGR), and twin anemia-polycythemia sequence (TAPS). TTTS and TAPS result from unbalanced shunting of blood between the two fetuses across the placental vascular anastomoses, whereas sFGR results mainly from unequal placental sharing. The outcome in TTTS has improved greatly in the past few decades, as a result of fetoscopic laser surgery, but the optimal management and outcome in sFGR and TAPS remain unclear and require further research. In this special edition, we will focus on various aspects of the delicate balance in MC twins, which can lead to three major complications: TTTS, sFGR, and TAPS.

## 1. Twin-Twin Transfusion Syndrome (TTTS)

The best treatment for twin-twin transfusion syndrome (TTTS) is fetoscopic laser coagulation of the vascular anastomoses, preferably using the Solomon laser technique [2]. Greimel et al. show that this surgical intervention is safe and rarely associated with maternal procedure-related complications [3]. Importantly, TTTS should not be viewed as a homogeneous disorder and may coexist with selective fetal growth restriction (sFGR) and/or twin anemia-polycythemia sequence (TAPS). In two large TTTS cohorts, Groene et al. and Tollenaar et al. report on the incidence and clinical consequences of these associated complications [4,5]. TTTS is also known to be related to an increased risk of cardiac and neurologic complications. In a systematic review, Gijtenbeek et al. report on the increased risk of acquired congenital heart diseases in TTTS, mainly attributed to the risk of right ventricular outflow tract obstruction [6]. These findings suggest that routine fetal echocardiogram should be performed in all monochorionic (MC) twins, including followup scans in the event of TTTS, and a postnatal cardiac evaluation in all TTTS survivors. In a large followup study in TTTS treated with laser surgery between 2011 and 2014, Spruijt et al. show that increased survival is (fortunately) not associated with increased risk of neurodevelopmental impairment [7]. In this study, low birth weight, growth restriction, and cerebral injury were identified as risk factors for poor neurodevelopmental outcomes. Importantly, neither the absence of severe prematurity nor the absence of cerebral injury precluded neurodevelopmental impairment, warranting routine long-term followup in all TTTS survivors.

Although the optimal treatment for TTTS, fetoscopic laser surgery, is nowadays clearly established, various issues remain to be clarified. One of the most important issues is a problem of organization regarding the centralization of this complex and technical intervention in highly specialized centers. There is currently an increasing number of small fetal therapy centers starting up in a deregulated manner. However, we should not forget that centralization of specialized care is of paramount importance to improve outcomes and ensure high quality of care. In TTTS, centralization of fetoscopic laser surgery treatment has been shown to lead to an improved learning curve, a reduction in complications, and improved survival [8]. Patients with TTTS in each country should have the right to claim the best possible treatment by a dedicated and experienced multidisciplinary team. At the end of the day, it is not about the individual fetal surgeon’s success rate or career, but only about ensuring the best possible management and outcome for patients with TTTS.

## 2. Selective Fetal Growth Restriction (sFGR)

In contrast to TTTS, very little is known about the optimal management and outcome in sFGR. More studies are required to determine if, how, and when these pregnancies should be treated antenatally. One of the most difficult decisions in perinatal medicine is when to induce delivery in sFGR. Delivering these babies at a too early age will lead to complications associated with severe prematurity (including neonatal mortality and cerebral injury), while delivering these babies too late may be associated with an increased risk of single (or double) fetal mortality and also cerebral injury (in case of single fetal demise). The management options in sFGR include expectant management or induced delivery, but also fetal surgical interventions such as fetoscopic laser coagulation of the anastomoses, and selective feticide. As shown by Druguet et al. in this edition, we should not underestimate the long-term psychological effects in parents that experienced fetal loss, particularly after selective feticide [9]. We should also not forget that management decisions in sFGR are currently not based on evidence but rely primarily on expert opinion and vary greatly between centers. Ideally, the optimal management strategy should be based not only on reliable data on chances of survival but also on chances of disease-free survival (i.e., without cerebral injury and long-term neurodevelopmental impairment). As shown by Groene et al. in a systematic review in this edition, knowledge on the long-term outcome in survivors with sFGR is unfortunately very limited [10]. We urgently need high-level evidence to guide us on the optimal management, including the best timing of delivery and reliable data to inform parents of the long-term outcome for both the growth-restricted infant and the appropriately-grown co-twin.

## 3. Twin Anemia-Polycythemia Sequence (TAPS)

Although the first description of TAPS dates now more than a decade ago [11], the management is still unclear and varies throughout the world. Diagnosis of TAPS can only be reached antenatally, through routine measurements of middle cerebral artery peak systolic velocity using Doppler ultrasound examination. In the United States, routine Doppler screening for the detection of TAPS is not recommended. Nicholas et al. argue that routine TAPS screening is a medically proven valuable resource that should be offered to all patients with MC twin pregnancies [12]. Although the optimal antenatal screening strategy remains to be established, an increasing body of work clearly shows that TAPS is associated with a high risk of perinatal mortality and morbidity and long-term sequelae, warranting increased scrutiny in the antenatal period as well as postnatally [13]. In a recent international collaboration between 17 fetal therapy centers, we were able to evaluate the management and outcome in the largest TAPS cohort (unpublished data), and we are currently in the process of presenting the results. This positive experience demonstrates that international multicenter cooperation can improve knowledge and serve as a base for future trials in MC twins with rare conditions.

In conclusion, a collaboration between fetal surgeons, neonatologists, and psychologists is crucial to continue improving the quality of care and outcome in complicated MC twins. In this endeavor, we should pay careful attention to centralizing this specialized care and involve twin parents’ organizations to determine what they think the research agenda should focus on. There is still room for improvement, especially in the cases of sFGR and TAPS. As long as these two fetuses remain connected through the vascular anastomoses, they remain highly vulnerable, just like to two equilibrists on a thin line. If one falls, both may fall. Specialists in complicated MC twin pregnancies throughout the world should join forces to help these babies survive (without complications) through research collaboration and centralization of care.
